# Treatment Options in Impacted Maxillary Canines: A Literature Review

**DOI:** 10.3390/dj13090433

**Published:** 2025-09-18

**Authors:** Saverio Ceraulo, Antonio Barbarisi, Beatrice Oliva, Sharon Moretti, Gianluigi Caccianiga, Dorina Lauritano, Roberto Biagi

**Affiliations:** 1 Department of Medicine and Surgery, University of Milano-Bicocca, 20100 Monza, Italy; 2 Fondazione IRCCS San Gerardo dei Tintori, 20900 Monza, Italy; 3 Department of Translational Medicine, University of Ferrara, 44121 Ferrara, Italy; 4 Department of Biomedical, Surgical and Dental Sciences, School of Dentistry, University of Milan, 20122 Milan, Italy; 5 UOC di Chirurgia Maxillo-Facciale e Odontostomatologia, Fondazione IRCCS Cà Granda Ospedale Maggiore Policlinico, Via della Commenda 10, 20122 Milan, Italy

**Keywords:** canine, impaction, maxillary, diagnosis, treatment

## Abstract

**Background**: Impaction of maxillary canines is a frequent clinical challenge in orthodontics. Early diagnosis is key to effective management. **Methods**: This narrative review included studies published from 2004 to 2024. An electronic search was conducted in PubMed, Scopus, and Google Scholar (September–November 2024), using predefined eligibility criteria. The selection and drafting were completed in the following months. Studies involving orthopedic, orthodontic, or surgical-orthodontic management of impacted maxillary canines were included. Case reports and procedures limited to avulsion or transplantation were excluded. **Results**: A total of 10 studies were analyzed, comprising 5529 patients, of whom 2530 met the criteria for treatment-specific analysis. Surgical exposure with orthodontic traction was the most frequent treatment (72%), followed by monitoring (12%), maxillary expansion (6%), and extractions (10%). Interceptive approaches were mainly applied in patients aged 7–18 years, with favorable outcomes especially before age 12. In adults, more invasive treatments were required, often with reduced success rates. **Conclusions**: Early diagnosis and interceptive extraction of deciduous canines reduce treatment complexity and improve success. Therapeutic outcomes are strongly influenced by patient age, tooth position, and angulation. A structured, radiographically guided approach, supported by the proposed decision-making flowchart, may optimize clinical outcomes. However, heterogeneity of included studies and lack of long-term follow-up limit the strength of available evidence.

## 1. Introduction

An impacted canine is defined as a tooth with complete root formation that fails to erupt due to obstruction by another tooth, bone, soft tissue, or pathology [1]. Epidemiologically, it is the second most commonly impacted tooth after the third molars. Impaction is more frequent in females, with a prevalence twice that of males. Furthermore, maxillary canine impaction is approximately ten times more common than mandibular impaction. In 85% of cases, the impaction is palatal, with a 2:1 ratio compared to buccal impaction, and approximately 8% of cases are bilateral. Impactions are often associated with agenesis or microdontia of the maxillary lateral incisors. Additionally, impacted canines are more frequently observed in individuals with skeletal Class II malocclusion [2,3].

The classification of impacted maxillary canines is based on several parameters, including buccal or palatal position, vertical, horizontal or oblique angulation, and spatial relationship to adjacent teeth.

Ericson and Kurol (1986) introduced a five-sector classification based on the position of the canine cusp tip in panoramic radiographs and defined the α-angle to quantify its inclination relative to the midline: interceptive extraction is most effective when the canine has a moderate angulation (20–30°) and is located in sectors 2–3; in severe cases (alpha angle >30°, sector 4), early surgical exposure with orthodontic traction is preferred to avoid further impaction and root resorption [4].

Peck & Peck (1995) proposed a transposition-based classification, including categories such as maxillary canine–first premolar and maxillary canine–lateral incisor [5].

Three-dimensional classification systems utilize CBCT to assess crown position in vertical, mesiodistal, and buccolingual planes, as well as associated pathologies such as root dilaceration or adjacent tooth resorption [6].

Radiographic diagnosis of impacted canines traditionally begins with two-dimensional panoramic and periapical radiographs. However, cone beam computed tomography (CBCT) offers a more precise three-dimensional evaluation, allowing accurate determination of the position of the impacted tooth, its relationship with adjacent structures, and the presence of root resorption. CBCT is especially indicated when 2D images are inconclusive [7].

Impacted canines may be associated with follicular cysts or, in rare cases, ameloblastomas. Follicular cysts can cause root resorption of neighboring teeth if untreated, while ameloblastomas—although rare—require histopathological diagnosis due to their aggressive and infiltrative behavior [8].

Contact between the impacted tooth and the cortical plate or incisive canal plays a critical role in surgical planning. [9].

Root dilaceration and proximity to the maxillary sinus complicate management of impacted canines. Dilacerated roots may hinder orthodontic traction or extraction, while sinus involvement raises the risk of sinus perforation during surgery [10].

Failure of resorption of the primary canine is an early indicator of impaction risk. Timely extraction of the primary canine—ideally between ages 8 and 10—may promote spontaneous eruption of the permanent canine and reduce the need for invasive treatment [11].

Regarding etiology, some authors highlight genetic factors, while others attribute impaction to the long eruptive pathway the canine must follow to reach its final position in the dental arch [12,13,14]. Maxillary canines develop high near the nasal bridge and migrate inferiorly and buccally, following the course of the nasolacrimal duct. This movement results from the pyramidal shape of the maxilla, which flares outward toward its lower portion. As the canine approaches the apex of the lateral incisor, it shifts direction and follows a vertical eruptive trajectory along the root of the lateral incisor until reaching the occlusal plane [1].

Therefore, any anomaly in the size, shape, angulation, or root dilaceration of the lateral incisor can interfere with this guidance and lead to unilateral canine impaction. Another theory suggests that crowding and lack of space are primary etiological factors, as in many cases, space creation in the arch results in the spontaneous eruption of the canines [15].

Based on the position of the canine relative to the alveolar process, impactions can be classified as palatal or buccal. About 85% of palatally impacted canines and 17% of buccally impacted ones have sufficient space for eruption. Buccally impacted canines tend to have a favorable vertical inclination, whereas palatally impacted canines often present with a horizontal orientation. Consequently, buccal impactions may erupt without surgery, while palatal impactions rarely do so due to the thickness of the palatal cortical bone [16].

There is general consensus that palatal and buccal impactions have distinct etiologies. The primary cause of buccal impaction is crowding. Regarding palatal impaction, two main theories exist: the guidance theory and the genetic theory [17]. According to the guidance theory, Miller and Bass observed a high prevalence of agenesis of the maxillary lateral incisor associated with palatal impaction and proposed that the canine lacks the eruptive guidance typically provided by the lateral incisor’s distal root surface. However, Miller argued that even a small or conical lateral incisor root should be sufficient to guide the canine [18].

Although a higher incidence of hypoplastic or conoid lateral incisors has been noted in patients with palatal impactions, many such cases present with normal lateral incisors, a fact that challenges the guidance theory [19].

The causes of impaction can be classified into systemic and local factors. Local factors are more prevalent and include:

- Arch lengthtooth size discrepancy
- Prolonged retention or early loss of the deciduous canine
- Abnormal position of the tooth germ
- Lateral incisor agenesis
- Alveolar clefts
- Ankylosis
- Cystic or neoplastic lesions
- Root dilaceration
- Iatrogenic or idiopathic causes

Crowding is widely recognized as one of the leading causes of both palatal and buccal canine impaction [20,21,22]. Systemic causes include various diseases, syndromes, and systemic conditions such as hypopituitarism, hypothyroidism, cleidocranial dysplasia, Down syndrome, achondroplasia, hypovitaminosis A or D, amelogenesis imperfecta, and osteoporosis [23].

The optimal time for evaluating the risk of impaction is during the early mixed dentition phase. Early diagnosis of dental anomalies may serve as a predictor for the development of further anomalies [24,25,26].

Initial clinical and radiographic evaluation is essential, with panoramic radiography being the preferred diagnostic tool [27]. Early detection is critical for successful interceptive treatment. Delayed diagnosis may result in complications such as crowding, root resorptions, or unfavorable horizontal eruption paths, necessitating more complex interventions [28].

Clinically, around ages 9–10, the canine bulge should be palpated bilaterally at the buccal and palatal levels. Non-palpability may suggest impaction. Moreover, persistence of the deciduous canine beyond 12–13 years of age without signs of mobility or presence of a palpable permanent canine may also indicate impaction. The morphology and position of the lateral incisors should be evaluated, as their distal tipping and rotation may be associated with palatal canine impaction [29].

Treatment options depend on clinical findings, canine position, and patient preference. They include observation, extraction, surgical exposure with orthodontic alignment, or surgical repositioning of the impacted tooth [30]. Interceptive extraction of the deciduous canine in patients aged 10–11 years with palatally displaced permanent canines has been shown to be effective, with diminished success in older patients (12–14 years) [31,32]. The likelihood of spontaneous eruption decreases as the canine crown becomes more mesially positioned [33].

Interceptive treatment often involves early extraction of the deciduous canine, rapid palatal expansion, or orthopedic traction systems to create space. These interventions reduce the risk of malocclusion and the need for more complex orthodontic treatment. Gaining space can alter environmental conditions and redirect the eruptive path [34,35].

If interceptive extraction fails due to late diagnosis, age, or unfavorable canine position, surgical exposure with orthodontic traction should be considered [36]. Surgical techniques may be categorized as open or closed based on the approach [37]. Orthodontic traction is particularly effective in growing patients, guiding the impacted canine into the arch. Potential complications include bone loss, root resorption, and gingival recession. In certain cases, micro-implants can provide skeletal anchorage, but they are contraindicated in horizontally impacted canines due to root movement risks [38].

Fixed orthodontic appliances with reinforced anchorage are often essential in complex cases to mitigate the adverse effects of traction forces [39]. Surgical risks include gingival recession, bone and attachment loss, ankylosis, vascular damage, and pulp necrosis. Treatment duration is typically around two years, requiring patient motivation and compliance [18]. Additional complications may include root resorption of adjacent teeth, dentigerous cysts, pain, swelling, internal resorptions, tooth migration, and space loss in the arch [1].

This literature review aims to identify the most appropriate treatment approach for impacted maxillary canines based on patient age, impaction position, and depth.

## 2. Materials and Methods

This review is a narrative review with structured elements. It is not a systematic review, and therefore PRISMA or PROSPERO registration was not applied.

**Figure 1:**
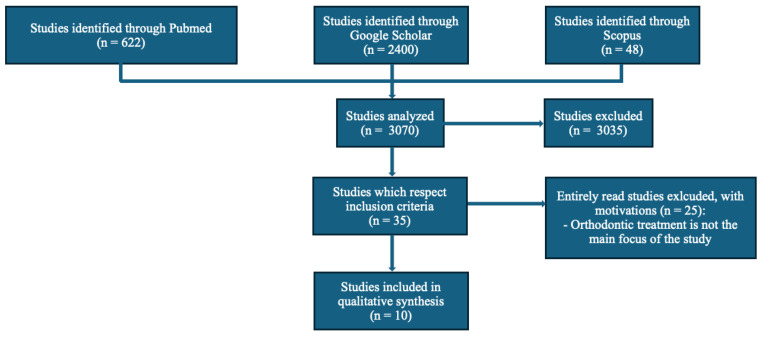
PRISMA flow diagram.

Inclusion criteria included studies concerning orthopedic, orthodontic or orthodontic-surgical treatment of impacted canines in the upper jaw. Studies adopting only avulsion, autotransplantation and intentional reimplantation as treatment options were excluded. Restrictions were applied regarding the publication date of the studies, excluding articles prior to 2004. Literature reviews, systematic reviews, randomized controlled trials and cross-sectional studies were included in the selection. Case reports and case series were excluded from the selection. No restrictions were applied regarding the language in which the study was published. An electronic search was conducted in the Pubmed (MEDLINE), Scopus and Google Scholar databases between September 2024 and November 2024. The selection, full-text reading, and drafting of the manuscript were carried out in the following months. The search strategy used was: “(maxillary OR upper impacted OR displaced canine) AND (orthodontic OR surgical-orthodontic treatment)”. The articles were initially selected by reading the titles and abstracts and, subsequently, those that met the eligibility criteria were read in full. In addition, a manual search was conducted of the references of the selected studies in order to examine all the articles in the area of interest. The data were collected in a table in order to catalog the following information: author and year of publication, type of study, objective of the study, type of treatment considered, number of studies analyzed, number of patients, age of patients, number of teeth considered, buccal/palatal impaction and position of the impacted tooth. Figure 1 shows the PRISMA flow diagram.

## 3. Results

The electronic search produced 3070 articles, of which 3035 articles were excluded because they did not concern the topic of interest, were duplicates, did not meet the inclusion criteria or contained one or more exclusion criteria. After this initial screening, 35 articles were selected to be read in full. Following in-depth reading, 25 articles were excluded because they did not have as their main focus the orthodontic treatment of impacted canines. Therefore, the studies included in the qualitative synthesis were 10. Table 1 shows the selected studies that reflect the inclusion criteria.

**Figure 2:**
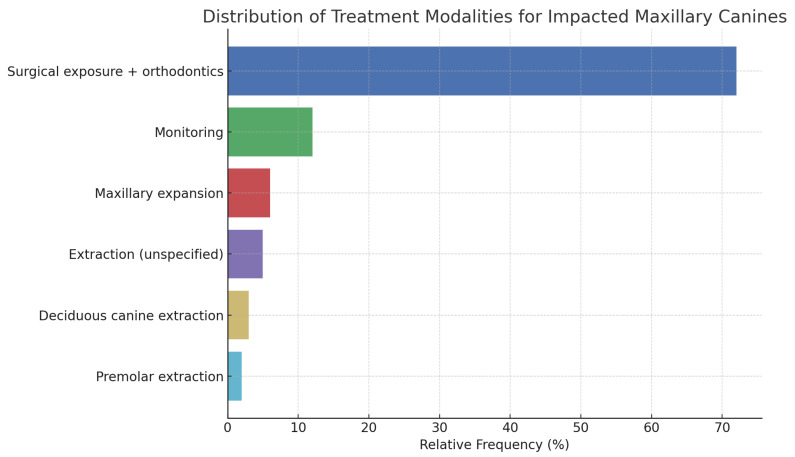
Distribution of relative frequencies of treatments.

Out of a total of 5529 patients analyzed across the studies included in Table 1, a sample of 2530 patients (45.75%) was selected for analysis. Patients from studies that did not specify the number of individuals undergoing specific treatments were excluded. Analysis of Figure 2 shows the following distribution of treatment modalities by relative frequency: Surgical exposure followed by orthodontic treatment is the most common approach, accounting for 72% of the sample. Monitoring is the second most frequent strategy, observed in 12% of cases. Extraction of deciduous canines and premolars were reported in only 3% and 2% of cases, respectively. An additional 5% of patients underwent unspecified extraction procedures, while maxillary expansion was employed in 6% of the sample. In summary, surgical exposure combined with orthodontic treatment clearly represents the predominant therapeutic approach, whereas other options, such as extraction of deciduous canines or premolars, are used far less frequently.

## 4. Discussion

### 4.1. Timing of Diagnosis

The optimal timing for early diagnosis is between 9 and 10 years of age, when the permanent upper canine begins its eruptive path. Early diagnosis, both clinical and radiographic [29], is optimally performed during the early mixed dentition stage, since a dental anomaly may indicate increased risk for subsequent problems [23]. OPT radiography is the method of choice for initial evaluation [42], and timely diagnosis is crucial for the success of interceptive treatment [43]. Clinically, around 9–10 years of age, the canine bossing should be palpated bilaterally with the index fingers, both at the buccal and palatal level [44]. If it cannot be palpated, impaction of the upper canine should be suspected [45]. Furthermore, the permanence of the deciduous canine beyond 12–13 years of age in the absence of tooth mobility and palpation of the canine boss is indicative of permanent canine impaction. The morphology of the permanent upper lateral incisors must also be evaluated in terms of position or angulation anomalies. In fact, palatal impaction of the canine can determine distal inclination and rotation of the upper lateral incisor. Clinical signs of canine impaction include delayed eruption of the permanent canine, prolonged retention of the deciduous canine, absence of the canine boss, presence of palatal boss, distal tipping of the lateral incisor crown [46,47].

### 4.2. Interceptive vs. Corrective Approaches

Treatment options depend on the clinical case, examination findings, and patient preferences, and include observation, extraction, surgical exposure with orthodontic traction, or surgical repositioning of the impacted tooth [1]. Interceptive extraction of deciduous canines in patients with palatally impacted permanent canines during mixed dentition has proven effective, particularly in younger patients (10–11 years), while success rates decline in older subjects (12–14 years) [48]. Surgical exposure with orthodontic traction is often required when the canine crown is more mesial, as the probability of spontaneous eruption decreases [49]. Interceptive treatment may also combine deciduous canine extraction with rapid palatal expansion and/or orthopedic traction to create adequate space for eruption, reducing the risk of future malocclusions and the need for complex orthodontic procedures [50,51]. Both palatal and buccal impactions can benefit from interceptive procedures, as these approaches help maintain or increase arch length [52,53,54]. Following extraction, space maintainers are recommended to prevent mesial migration of molars, especially in older patients [55]. If interceptive treatment fails—because of age, late diagnosis, resorption, or unfavorable canine position—surgical exposure with subsequent orthodontic traction becomes necessary [56].

### 4.3. Surgical vs. Orthodontic Strategies

Surgical exposure, with or without orthodontic traction, encompasses various techniques that can be differentiated into open and closed depending on the surgical approach. Many studies have investigated the influence of the surgical technique on the outcome of the treatment: the open technique seems to be better to the closed technique in terms of reduction in time for initial alignment and reduction in the risk of ankylosis [57].

Orthodontic traction is the most appropriate treatment in cases diagnosed early, as the subjects are still in the growth phase. It consists of surgical exposure of the impacted tooth and subsequent traction to guide and align it in the arch. The most common complications of surgery are bone loss around the extraction site, root resorption and gingival recessions [58]. Risks related to surgery are represented by gingival recessions, bone loss and loss of gingival attachment, especially if it is not ensured that the canine erupts or is positioned in the keratinized gingiva [59]. If the canine has to be moved from a considerable distance, ankylosis is a real possibility, as is loss of vascular supply and, consequently, pulp necrosis. Treatment usually requires 2 years, and it is important that the patient is motivated and cooperative [60].

### 4.4. Complications

Regarding complications, the main ones are represented by the formation of root resorptions of adjacent permanent teeth, dentigerous cysts, pain, swelling, internal resorptions, migration of adjacent teeth and loss of space in the arch [1].

The management of impacted maxillary canines requires careful consideration of various factors to determine the most appropriate surgical and orthodontic approaches. The decision to use an open or closed surgical exposure technique depends largely on the position of the impacted tooth and periodontal factors. Open exposure is typically recommended for labially impacted canines with adequate keratinized tissue, allowing for easier orthodontic traction and better visualization during surgery. In contrast, closed techniques are more suitable for palatally displaced canines, helping preserve mucosal integrity, reducing postoperative discomfort, and potentially improving periodontal outcomes [61,62]. Recent prospective studies have further confirmed that the choice between open and closed surgical exposure significantly influences healing time, patient comfort, and the long-term periodontal status, with closed exposure showing advantages in preserving gingival attachment and reducing recession risk in palatal impactions [63,64]. Therefore, individual patient anatomy, tooth position, and periodontal health must be evaluated comprehensively to optimize treatment success and minimize complications.

### 4.5. Decision-Making Tools

When exposing labially impacted teeth, the use of an apically repositioned flap is particularly indicated to maintain or enhance the zone of attached gingiva, thereby preventing future mucogingival issues and supporting better long-term periodontal health [65]. The path taken for orthodontic traction significantly influences treatment efficiency; in cases of palatal impaction, initiating traction toward the palate before redirecting it toward the arch can minimize damage to adjacent teeth and improve eruption control [66]. Proper anchorage is essential throughout traction, and the use of skeletal anchorage devices, such as TADs, can stabilize anchorage units and improve the precision of tooth movement by avoiding unwanted reciprocal forces [38,67].

One of the most serious risks during canine disimpaction is root resorption of adjacent teeth, which can be prevented not only by ensuring that roots are spatially separated from the impacted canine, but also by carefully calibrating the magnitude and direction of applied orthodontic forces [68,69].

Orthodontic auxiliaries such as springs, levers, and elastics also play a vital role, and their judicious selection based on individual clinical situations can accelerate canine eruption and improve biomechanical control [70]. In cases requiring accelerated tooth movement, corticotomy-assisted techniques may be considered; these facilitate bone remodeling and have proven effective in reducing overall treatment time, particularly in adults [71].

The minimally invasive corticotomy-assisted orthodontic treatment demonstrated a faster velocity of traction movement for palatally impacted canines and reduced treatment duration without causing significant adverse dentoalveolar changes [72,73].

Corticotomy-assisted orthodontic therapy enhances bone remodeling through the regional acceleratory phenomenon, improving periodontal health and facilitating quicker orthodontic tooth movement with less risk of root resorption [74].

Laser-assisted exposure is a valuable adjunct in the management of impacted canines, as it not only enhances healing and minimizes surgical trauma but also improves soft tissue management and access, thereby supporting more efficient orthodontic traction. Recent studies have demonstrated that the photobiomodulating action of diode lasers can facilitate the disinclusion of palatally impacted canines, promoting better postoperative outcomes and patient comfort [75,76]

Special care must be taken when treating patients with partial fixed appliances, as changes in the inclination of lateral incisors may inadvertently influence the eruption path of adjacent impacted canines, potentially causing deviation or retention of the tooth; vigilant control of incisor position is necessary to prevent such complications [70].

### 4.6. Age-Related Treatment Outcomes

Analyzing the data collected for this study, different treatment modalities emerge for patients with impacted maxillary canines, which vary not only according to the position of the inclusion (palatal or buccal), but above all in relation to the age of the patient at the time of diagnosis.

**Figure 3:**
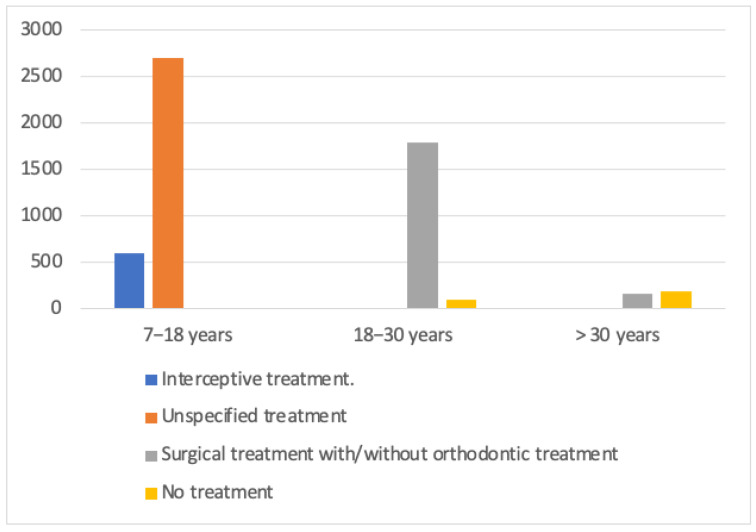
View of treatments by age.

The data extracted from the ten selected studies are reported in Table 1, with a total sample of 5529 patients showing that the majority of these (3299 patients) belong to the age group between 7 and 18 years, for which the treatment chosen by the clinician is the interceptive one. In this group, 407 patients obtained the resolution of the inclusion by extraction of the deciduous canines, 151 by expansion of the maxillary, and 40 thanks to the extraction of the premolars. In the remaining 2701 patients, the interceptive treatment adopted was not specified. Surgical treatment or simple monitoring were not included in this age group. For patients aged 18 to 50 years, more invasive treatments were adopted. In the 1885 patients aged 18 to 30 years, 91% received a combined treatment of surgical exposure and orthodontics, 5% were only monitored, and 4% were treated with extraction therapy. Among patients aged over 30 years, 53% did not receive any active treatment, limited to monitoring, while 31% received orthodontic and surgical treatment, and 16% were treated with extraction therapy (Figure 3). The analysis highlights the importance of early diagnosis of maxillary canine impaction, as this allows to reduce the operative difficulties during the intervention. A late diagnosis in fact leads to an increase in the complexity and invasiveness of the treatment to be undertaken.

### 4.7. Quality of Evidence and Risk of Bias

The extended table below (Table 2) provides a detailed risk of bias analysis of 10 selected articles on impacted maxillary canines. It includes study design, bias risk level, and also evaluates sample size, outcome measures, limitations stated by the authors, and ethical approval to better determine each study’s evidential value.

Among the highest-quality sources, the systematic review by Benson et al. [32] stands out for its rigorous methodology and inclusion of randomized controlled trials (RCTs). This review confirmed that non-surgical interventions may promote spontaneous eruption in select cases but also highlighted the limited volume and heterogeneity of available trials. RCTs by Bazargani et al. [36] and Hadler-Olsen et al. [31] further substantiated the benefits of interceptive extraction, showing significant improvements in eruption outcomes, while Naoumova and Kjellberg [33] provided radiographic criteria to optimize timing.

In contrast, observational and cross-sectional studies—such as those by Brézulier et al. [41] and Hasan et al. [1]—offered useful insights but suffered from inherent methodological limitations, including selection bias and lack of longitudinal validation. Narrative reviews [13,23] added descriptive overviews but, due to high risk of bias, contribute less to evidence-based decision-making.

Overall, only three studies [31,32,36] were judged at low risk of bias, providing the most robust and clinically actionable evidence. The majority, however, presented moderate to high bias, limiting external validity. This uneven methodological quality underscores the need for additional multicenter RCTs with standardized outcomes and long-term follow-up. Until then, current conclusions—particularly regarding interceptive treatment timing—should be interpreted with caution.

### 4.8. Proposed Clinical Decision-Making Flowchart

**Figure 4:**
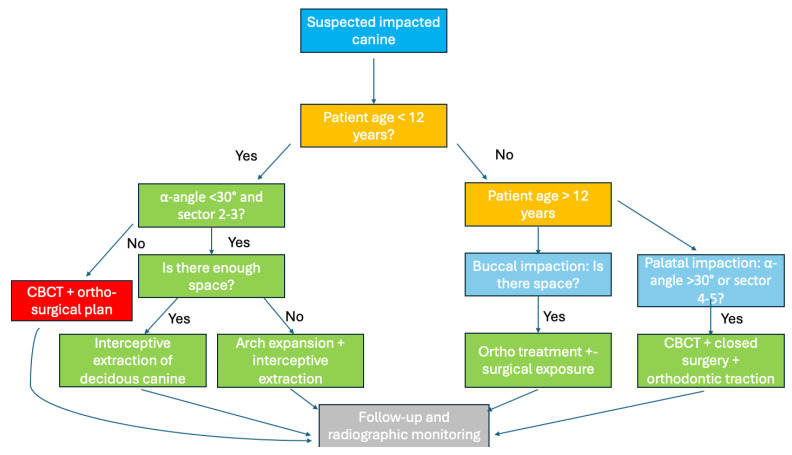
Flowchart for decision-making in the management of impacted maxillary canines. The algorithm integrates four critical variables: patient age (<12 or ≥12 years), impaction location (buccal vs. palatal), α-angle (>30° = increased difficulty), and cusp position according to Ericson & Kurol’s classification (sectors 4–5 = higher risk). These factors guide clinicians toward the most appropriate intervention: interceptive extraction in early-diagnosed favorable cases, maxillary expansion when space is inadequate, or surgical-orthodontic planning for complex scenarios.

Based on the treatment outcomes reviewed in this article and the correlation between therapeutic success and factors such as patient age, canine position, and α-angle, a clinical decision-making flowchart has been developed (Figure 4). This tool is designed to assist clinicians in the early diagnosis and management of impacted maxillary canines. The algorithm integrates four critical variables:

- Patient age (<12 or ≥12 years)
- Canine impaction location (buccal vs. palatal)
- αangle (with >30° indicating increased difficulty)
- Cusp position according to Ericson & Kurol’s sectors (sector 4–5 = higher risk)

These factors are used to guide clinicians toward the most appropriate intervention pathway:

- Interceptive extraction in favorable earlydiagnosed cases
- Maxillary expansion when space is inadequate
- Surgicalorthodontic planning in more complex scenarios
- CBCT imaging when conventional radiographs are inconclusive or severity indicators are high

This visual algorithm helps standardize the clinical workflow and supports a more objective and reproducible decision-making process. It offers a bridge between evidence synthesis and practical application, adding a valuable original element to the current literature.

### 4.9. Limitations

The main limitations of this narrative review (Table 3) include the heterogeneity of included studies, the small number of high-quality randomized controlled trials, potential publication bias, and the lack of long-term follow-up in most trials. These factors reduce the strength of the conclusions and highlight the need for further high-quality research.

## 5. Conclusions

Early diagnosis and interceptive extraction of deciduous canines are confirmed as the most effective strategies to reduce treatment complexity for permanent canine impactions. Treatment planning must integrate age, impaction position, α-angle, and sector classification.

The proposed clinical decision-making flowchart translates current evidence into a structured and practical tool, helping clinicians identify when interceptive treatment is sufficient and when surgical-orthodontic planning is required.

Nevertheless, the heterogeneity of included studies and the scarcity of high-quality RCTs limit the strength of the available evidence. Future well-designed, multicenter trials are needed to validate standardized treatment protocols.

## Figures and Tables

**Table 1:** Papers selected for this search.

Author/Year	Study Design	Sample Size	Age Range	Intervention	Outcome	Impaction Type
Leonardi et al., 2004 [40]	RCT	75	7–13 years	Extraction of deciduous canines	Sectors 2–5	Not specified
Brézulier et al., 2023 [41]	Cross-sectional study	151	7–13 years	Maxillary expansion	Improved eruption path	Not specified
Bazargani et al., 2014 [36]	RCT	24	10–14 years	Extraction of deciduous canines	Spontaneous eruption in favorable cases	Sectors 2–5
Hadler-Olsen et al., 2020 [31]	RCT	32	9–13 years	Extraction of deciduous canines	Favorable eruption outcomes	Palatal, Sectors 2–4
Naoumova & Kjellberg, 2018 [33]	RCT	67	10–13 years	Interceptive extraction	Timing improved treatment success	Palatal
Benson et al., 2021 [32]	Systematic review	199	9–14 years	Extraction/Monitoring	Higher eruption rates in early diagnosis	Palatal
Grisar et al., 2021 [27]	Systematic review	1247	9–18 years	Interceptive and orthodontic	Relationship between canine position & success	Both
Hasan et al., 2022 [1]	Cross-sectional	90	18–30 years	Monitoring/Extraction	Variable	Mixed
Aquino-Valverde et al., 2021 [23]	Narrative review	39	–	Microimplants/Anchorage	Orthodontic alignment support	Not specified
Grybienė et al., 2019 [13]	Narrative review	23	–	Interceptive + orthodontic	Summary of diagnostic strategies	Both

**Table 2:** Bias analysis of the papers included in the work.

Author/Year	Study Design	Bias Risk Level	Tool Used	Sample Size	Outcome Measures	Limitations	Ethical Approval
Aquino-Valverde et al., 2021 [23]	Narrative review	High	None	–	Descriptive only	No critical appraisal	Not mentioned
Benson et al., 2021 [32]	Systematic review	Low	Cochrane RoB 2.0	4 RCTs	Spontaneous eruption	Limited number of studies	Not stated
Bazargani et al., 2014 [36]	RCT	Low	Not stated	67	Eruption success	Single-center study	Not mentioned
Brézulier et al., 2023 [41]	Cross-sectional	Moderate	None	102	Presumptive signs of impaction	No causal inference	Ethical approval obtained
Grisar et al., 2021 [27]	Systematic review	Moderate	Not stated	15	Initial canine position vs. outcome	Heterogeneous studies	Not mentioned
Grybienė et al., 2019 [13]	Narrative review	High	None	–	Descriptive	No bias analysis	Not mentioned
Hadler-Olsen et al., 2020 [31]	RCT	Low	Not reported	240	Single vs. double extraction	Blinding not stated	Not mentioned
Hasan et al., 2022 [1]	Cross-sectional	Moderate	None	60	CBCT indicators	Selection bias	Not mentioned
Naoumova & Kjellberg, 2018 [33]	RCT	Low	Not stated	67	Timing of extraction	Limited generalizability	Not mentioned
Ristaniemi et al., 2022 [28]	Observational	Moderate	None	142	Radiographic features	Retrospective design	Not applicable

**Table 3:** Limitations of the study.

Limitation	Evidence Gap	Suggested Future Research
Few high-quality RCTs	Lack of robust long-term evidence	Conduct large multicenter randomized controlled trials
Heterogeneity in study designs and populations	Difficult to compare outcomes	Standardize inclusion criteria and outcome measures
Short follow-up periods	Uncertainty about long-term stability	Longitudinal studies with ≥5–10 years follow-up
Limited bias assessment tools	Unclear reliability of conclusions	Apply standardized tools (RoB 2.0, ROBINS-I)
Prevalence of narrative reviews	Weak evidence base for guidelines	Promote systematic reviews with registered protocols

## Data Availability

The authors declare no conflicts of interest.
